# Comparative Accuracy Assessment of Large Language Models in Cardiothoracic Anesthesia: A Performance Analysis of Claude and ChatGPT-4 on Subspecialty Board-Style Questions

**DOI:** 10.7759/cureus.88591

**Published:** 2025-07-23

**Authors:** Jordan Beil, Ishan Aggarwal, Mallikarjuna Devarapalli

**Affiliations:** 1 Anesthesiology and Perioperative Medicine, Augusta University Medical College of Georgia, Augusta, USA

**Keywords:** artificial intelligence, cardiothoracic anesthesia, clinical decision support, large language models, medical education

## Abstract

Background

The integration of artificial intelligence (AI) into healthcare has accelerated rapidly since the public release of ChatGPT (Open AI, San Francisco, California, United States) in 2022. While large language models (LLMs) have demonstrated proficiency in general medical knowledge and licensing examinations, their performance in specialized medical subspecialties remains largely unexplored.

Objective

The objective of this study was to compare the accuracy of two prominent LLMs, Claude (Anthropic PBC, San Francisco, California, United States) and ChatGPT-4, in answering cardiothoracic anesthesia board-style questions and evaluate their potential for clinical decision support in this subspecialty.

Methods

We developed a Python-based framework to systematically evaluate LLM performance on 100 custom multiple-choice questions covering cardiothoracic anesthesia topics including arrhythmia management, electrophysiology procedures, pacemaker programming, and perioperative complications. Questions were presented to both Claude and ChatGPT-4 via their respective application programming interface (APIs), with responses compared against expert-validated correct answers. The primary outcome was overall accuracy percentage for each model.

Results

Claude achieved 32% accuracy (32/100 questions), while ChatGPT-4 achieved 23% accuracy (23/100 questions), representing a 9% point difference (p < 0.05). Both models performed below the threshold typically considered acceptable for clinical decision-making (≥80%). Performance varied across question domains, with both models demonstrating marked difficulty in questions requiring complex electrophysiological reasoning and visual data interpretation (e.g., ECG and imaging-based cases).

Conclusions

Current LLMs demonstrate limited accuracy in subspecialty-level cardiothoracic anesthesia knowledge, highlighting the need for specialized training datasets and model refinement before clinical implementation. These findings underscore the importance of subspecialty-specific validation before deploying AI tools in specialized medical domains.

## Introduction

The landscape of healthcare technology has been fundamentally transformed since the public release of ChatGPT (Open AI, San Francisco, California, United States) in November 2022, catalyzing unprecedented interest in applying artificial intelligence (AI) to clinical practice [1]. Healthcare applications have rapidly emerged across multiple domains, including AI-powered clinical documentation systems, radiology interpretation tools, and decision support platforms, demonstrating the healthcare community's enthusiasm for integrating large language models (LLMs) into routine practice [2,3].

LLMs have shown remarkable proficiency in general medical knowledge assessment, with ChatGPT demonstrating passing performance across all three United States Medical Licensing Examination (USMLE) steps [4]. Similarly, Claude (Anthropic PBC, San Francisco, California, United States), another prominent LLM released in 2023, has shown promise in generating clinical documentation and answering board-style questions in various medical specialties [5,6]. However, these encouraging results in general medical knowledge do not necessarily translate to competency in specialized subspecialty domains.

Anesthesiology, particularly cardiothoracic anesthesia, presents unique challenges for AI systems due to its reliance on complex physiological reasoning, real-time decision-making, and integration of multiple data sources including electrocardiographic interpretations, hemodynamic monitoring, and imaging studies. The subspecialty of cardiothoracic anesthesia demands expertise in cardiac electrophysiology, mechanical circulatory support, perioperative echocardiography, and management of complex arrhythmias-areas that may challenge current LLM capabilities. For example, one study displayed the importance of adequately controlling pulse pressure during coronary artery bypass grafts and its association with perioperative renal dysfunction or failure [7]. 

Despite growing interest in AI applications within anesthesiology, limited research exists comparing the performance of different LLMs in anesthesia subspecialties [8]. Previous studies have raised concerns about AI performance in anesthesiology more broadly, suggesting that the complexity of perioperative decision-making may exceed current AI capabilities [9]. Recent investigations have explored the potential of domain-adapted models, such as Hypnos, which is specifically tuned for anesthesiology tasks, though its performance remains under active evaluation [10]. Furthermore, no studies have specifically evaluated LLM performance in cardiothoracic anesthesia, a subspecialty requiring integration of advanced cardiovascular physiology, cardiac surgical considerations, and specialized monitoring techniques.

The present study addresses this knowledge gap by systematically comparing the accuracy of two leading LLMs, Claude and ChatGPT-4, on a comprehensive set of cardiothoracic anesthesia board-style questions. Our primary objective was to evaluate whether current LLMs demonstrate sufficient accuracy for potential clinical decision support in this specialized domain. Secondary objectives included identifying patterns of performance across different question categories and developing a reproducible framework for subspecialty-specific AI evaluation.

## Materials and methods

Study design and setting

This comparative accuracy assessment was conducted using a standardized evaluation framework developed specifically for assessing LLM performance on medical subspecialty content. The study protocol was designed to simulate board examination conditions while maintaining reproducibility and objectivity in performance measurement. To ensure objectivity, questions were presented in a standardized format without additional context, and responses were collected and analyzed programmatically to minimize human intervention and bias. No randomization or blinding was applied, as models were evaluated deterministically via API queries.

Question development and validation

A comprehensive question bank of 100 multiple-choice questions was developed covering core competencies in cardiothoracic anesthesia, including (i) Cardiac electrophysiology and arrhythmia management (n=35), (ii) Pacemaker and implantable cardioverter-defibrillator programming (n=25), (iii) Perioperative complications and management (n=20), (iv) Pharmacology of antiarrhythmic agents (n=12), (v) Mechanical circulatory support devices (n=8).

Questions were designed to mirror the format and complexity of cardiothoracic anesthesia board examinations, with each question containing three to four answer choices. The questions used in this study were adapted from a widely used, published board preparation textbook in cardiothoracic anesthesia, which reflects current standards in the field. This resource was selected to ensure clinical relevance, consistency, and alignment with established educational objectives. Response accuracy was determined programmatically. The correct answer for each question was predefined based on the textbook source, and model outputs were compared automatically against this reference using a structured matching algorithm. This eliminated subjectivity in scoring and ensured consistent evaluation across all model responses. Questions requiring image interpretation (n=15) were noted separately, as current LLM APIs primarily process text-based inputs.

Technical implementation

A Python-based evaluation framework was developed to ensure standardized question presentation and response collection. The system architecture components are detailed in Table 1, which outlines the four primary modules and their respective functions within the evaluation pipeline. Further details on the system architecture and implementation specifications can be found in Appendices A and B.

**Table 1:** System architecture components Statistical analysis performed using Fisher's exact test for proportion comparisons.

Component	Description/Function
Data Processing Module (data_processing.py)	Managed question parsing, API interactions, and response caching to ensure consistent data handling throughout the evaluation process
Analysis Module (analysis.py)	Calculated accuracy metrics and performed statistical comparisons between models using standardized statistical methods
Visualization Module (visualization.py)	Generated performance comparison charts and summary reports for data presentation and analysis visualization
Orchestration Module (main.py)	Coordinated the complete evaluation pipeline, managing workflow execution and ensuring proper sequencing of all system components

In this study, we did not encounter any prompt failures, connection issues, or application programming interface (API) errors during data collection. Additionally, the design of the study did not permit retry attempts. Each model was given a single opportunity to respond to each prompt to ensure standardization and avoid introducing variability through retries. 

LLM evaluation protocol

Each question was presented to both Claude and ChatGPT-4 using standardized prompts through their respective APIs. The prompt format was: "Please answer the following multiple-choice question. Provide only the letter of the single best answer (A, B, C, D, etc.)”. To ensure consistency and minimize potential bias, questions were presented in an identical format to both models with no additional context or hints provided beyond the question text. Both Claude and ChatGPT-4 were accessed via their free, publicly available versions at the time of evaluation. These models were selected because they represent publicly accessible large language models commonly used in clinical, educational, and research settings. Responses were collected sequentially with a one-second interval between API calls, and all responses were cached to enable reproducible analysis. Sample questions and corresponding model answers can be found in Appendix C.

The risk of prompt injection was minimal in our study, as all questions were sourced from a standardized textbook and did not involve any user-generated or model-generated content that could introduce adversarial inputs. Hallucination detection was not necessary, as responses were programmatically parsed and evaluated solely based on whether they matched the correct letter choice. While LLMs are trained on publicly available web data, they can sometimes hallucinate and generate incorrect or fabricated information when they cannot retrieve a reliable answer from their internal knowledge base or simulated web-like recall. However, the strict answer formatting and reliance on a fixed question set in this study helped mitigate that risk.

Statistical analysis

The primary outcome was the overall accuracy percentage for each LLM, calculated as the number of correct responses divided by the total questions answered. Secondary analyses included category-specific accuracy comparisons, question difficulty analysis based on both models' performance, and inter-model agreement using Cohen's kappa coefficient. Cohen’s kappa measures agreement between two raters or models beyond chance, with values ranging from -1 (less than chance) to 1 (perfect agreement); commonly interpreted as slight (0.01-0.20), fair (0.21-0.40), moderate (0.41-0.60), substantial (0.61-0.80), and almost perfect (0.81-1.00) agreement. Statistical significance was assessed using Fisher's exact test for proportion comparisons, with p < 0.05 considered significant. All analyses were performed using Python 3.9 (https://www.python.org/downloads/release/python-390/) with pandas (https://pandas.pydata.org/) and scipy (https://scipy.org/) to ensure reproducible and standardized statistical evaluation. Further statistical analysis code is provided in Appendix D.

Ethical considerations

This study involved no human subjects and utilized only publicly available AI models. Questions were original content developed specifically for this research and did not include copyrighted material. No patient data or protected health information was used in the evaluation process.

It is acknowledged that the performance of freely available LLMs may be influenced by commercial strategies, including differential optimization to attract users. For instance, Claude, having launched after ChatGPT, may exhibit enhanced accuracy in its free version as part of a competitive positioning strategy. This introduces a potential confounding factor when comparing model performance. To address this, all models were evaluated using the same publicly accessible, free-tier settings available to the general public at the time of the study. No proprietary or premium APIs were used. Nonetheless, future studies should aim to reduce this variability by using standardized access tiers or controlled research environments to improve fairness and generalizability.

To reinforce ethical AI practices and promote model reproducibility, all prompts, evaluation criteria, and outputs were documented in detail. This ensures transparency in the methodology and enables independent replication of the study. While the underlying architectures of the LLMs remain proprietary, care was taken to use publicly known version identifiers and access methods to support reproducibility. The study aligns with emerging best practices in responsible AI research, including transparency, fairness, and accountability.

## Results

Overall performance comparison

Both LLMs completed the full 100-question assessment without technical failures or incomplete responses. Claude achieved an overall accuracy of 32% (32/100 questions correct), while ChatGPT-4 achieved 23% accuracy (23/100 questions correct). This nine-percentage-point difference was statistically significant (p = 0.042, Fisher's exact test). The overall performance summary is presented in Table 2, which demonstrates the comparative accuracy metrics and statistical significance of the observed differences between the two language models.

**Table 2:** Summary of overall performance Statistically significant (p < 0.05); Statistical analysis performed using Fisher's exact test.

Metric	Claude	Chat GPT-4	Difference	P-Value
Correct Answers	32/100	23/100	+9	0.042
Accuracy (%)	32%	23%	+9%	0.042
95% CI	23.1-41.8%	15.2-32.4%	-	-

Category-specific performance analysis

Performance varied significantly across content categories, with both models demonstrating consistent patterns of strength and weakness. The category-specific accuracy comparison is detailed in Table 3, which illustrates the performance variations across different subspecialty domains and highlights areas where both models encountered particular challenges. Further statistical analysis results are provided in Appendix E.

**Table 3:** Category-specific accuracy comparison

Category	Questions (n)	Claude Accuracy	ChatGPT-4 Accuracy	Difference
Cardiac Electrophysiology	35	34.3% (12/35)	25.7% (9/35)	+8.60%
Pacemaker Programming	25	36.0% (9/25)	24.0% (6/25)	+12.00%
Perioperative Complications	20	30.0% (6/20)	20.0% (4/20)	+10.00%
Antiarrhythmic Pharmacology	12	25.0% (3/12)	16.7% (2/12)	+8.30%
Mechanical Support Devices	8	25.0% (2/8)	25.0% (2/8)	+0.00%

Question difficulty analysis

Questions were stratified by combined model performance to identify particularly challenging content areas. High difficulty questions (0-25% combined accuracy) comprised 45 questions, moderate difficulty questions (26-50% combined accuracy) included 35 questions, and lower difficulty questions (>50% combined accuracy) encompassed 20 questions. Both models struggled most with questions requiring integration of multiple physiological concepts, interpretation of electrocardiographic findings, complex pharmacological reasoning, and device programming algorithms. Question categories and their distribution are detailed in Appendix F.

Inter-model agreement

Cohen's kappa coefficient for inter-model agreement was 0.23 (95% CI: 0.08-0.38), indicating fair agreement between models. Both models provided identical answers on 58% of questions (58/100), though this included both correct and incorrect concordant responses.

Image-based question performance

Of the 15 questions referencing electrocardiographic images or device tracings, both models performed poorly despite receiving detailed textual descriptions. Claude achieved 13.3% accuracy (2/15) while ChatGPT-4 achieved 6.7% accuracy (1/15). This suggests that even with comprehensive textual descriptions, the absence of visual data significantly impairs performance on image-dependent questions.

## Discussion

Principal findings 

This study represents the first systematic evaluation of LLM performance in cardiothoracic anesthesia, revealing significant limitations in current AI capabilities for this subspecialty. Both Claude and ChatGPT-4 demonstrated accuracy levels well below the threshold typically considered acceptable for clinical decision support (≥80%), with Claude showing modest but statistically significant superior performance. The overall accuracy rates of 32% (Claude) and 23% (ChatGPT-4) are substantially lower than the 60-85% accuracy these models have demonstrated on general medical licensing examinations [4,9]. This performance gap highlights the critical distinction between broad medical knowledge and subspecialty expertise, suggesting that current LLMs may have significant limitations when applied to specialized clinical domains.

Our findings align with a recent comprehensive scoping review by Lee et al. (2024), which analyzed 142 articles on LLM evaluation in medical fields [11]. Their systematic analysis revealed that LLM evaluation in medicine primarily falls into two categories: test examinations (37.3%) and evaluation by medical professionals (56.3%), with most studies utilizing 100 or fewer questions (29.0%). Importantly, their review highlighted that only 24.2% of studies performed repeated measurements and merely 12.9% employed prompt engineering techniques, suggesting that methodological rigor in LLM medical evaluation remains inconsistent across the literature. Our study contributes to this growing body of evidence by providing subspecialty-specific validation using a standardized, reproducible framework.

Subspecialty-specific performance limitations

The poor performance observed in our study reflects broader challenges in AI application to medical subspecialties. Recent research by Bogon et al. (2024) in their comprehensive review of AI in anesthesiology noted that while AI techniques have proven effective across various medical disciplines, anesthesia presents unique challenges due to its reliance on real-time decision-making, complex physiological reasoning, and integration of multiple data sources [12]. Their analysis identified specific applications where AI shows promise in anesthesiology, including preoperative risk assessment, intraoperative monitoring, and postoperative outcome prediction. However, they emphasized that successful implementation requires careful validation and integration with existing clinical workflows.

The subspecialty-specific limitations observed in our study are consistent with findings from other specialized medical domains. Multiple studies referenced in the Lee et al. scoping review demonstrated variable performance across different subspecialties, including neurosurgery board examinations, ophthalmology assessments, and radiation oncology physics evaluations [11]. This pattern suggests that the performance gap between general medical knowledge and subspecialty expertise may be a generalizable limitation of current LLMs rather than a specific deficiency in cardiothoracic content.

One of the key caveats in this study comparing large language models such as ChatGPT and Claude is that these are primarily pre-trained, general-purpose models. As their names suggest, they are designed to generate text based on broad training data, rather than being tailored for highly specialized domains. Their limited exposure to subspecialty-level multiple-choice questions-particularly in complex fields like cardiothoracic anesthesia-likely contributed to the lower accuracy observed in the study. This was reflected in both models’ inability to consistently answer these questions correctly.

Current open-access models like GPT-4, Claude, DeepSeek (Hangzhou DeepSeek Artificial Intelligence Basic Technology Research Co., Ltd, Hangzhou, Zhejiang, China), and LLaMA (Meta AI, New York, United States) are trained on generalized datasets and are not fine-tuned explicitly for subspecialty medical content. As a result, they struggle with nuanced clinical reasoning and domain-specific knowledge. To improve their performance in such settings, these models would need to be fine-tuned on targeted datasets that reflect the complexity and specificity of the subspecialty content.

Multimodal AI and clinical decision-making implications

The poor performance on image-based questions in our study (Claude: 13.3%, ChatGPT-4: 6.7%) highlights a critical limitation of current text-only LLM interfaces for subspecialties requiring extensive visual data interpretation. This finding is particularly relevant given recent advances in multimodal AI capabilities. Banerji et al. (2025) argued that multimodal AI has significant potential for clinical practice because clinical decisions are usually based on information from multiple sources [13]. However, they emphasized that unlike most developed multimodal AI workflows, clinical medicine is both a dynamic and interventional process where clinicians continually learn about patient health and act accordingly as data is collected.

The authors' systematic review revealed that only 22% of AI studies involved clinicians throughout development, while 82% involved clinicians specifically in the deployment phase only [13]. This lack of clinician involvement throughout the development process is particularly concerning for subspecialty applications like cardiothoracic anesthesia, where domain expertise is essential for appropriate model design and validation. Their findings suggest that detachment of clinicians from the development process is a driver of mistrust, while studies with high clinician involvement throughout development often benefit from high clinician confidence in the deployed tool.

Clinical implementation and validation requirements

The implications of our findings extend beyond academic interest to practical considerations for clinical implementation. Current evidence suggests that successful AI deployment in healthcare requires robust validation frameworks specifically designed for medical subspecialties. The poor performance observed in our study underscores the need for subspecialty-specific validation before deploying AI tools in specialized clinical areas, as recommended by emerging best practices in healthcare AI implementation.

Recent research has emphasized that AI validation in medical subspecialties requires consideration of multiple factors beyond simple accuracy metrics. These include algorithm validation, workflow integration, user-centric design principles, and continuous monitoring to ensure efficacy and usability in real-world clinical settings [14]. The development of our Python-based evaluation framework represents a step toward standardized subspecialty validation, providing a reproducible approach that can be adapted to other specialties and question formats.

Comparison with existing literature and performance benchmarks

Our findings align with previous research suggesting limitations in AI performance within anesthesiology subspecialties. Shay et al. (2023) reported similar concerns about ChatGPT's performance on general anesthesiology board questions, though differences in question content and evaluation methodology limit direct comparison [9]. The performance gap between general medical knowledge and subspecialty expertise observed in our study echoes findings from other specialized medical domains, suggesting this may be a generalizable limitation of current LLMs.

The broader literature on AI in anesthesiology reveals both promising applications and significant limitations. Bogon et al. (2024) identified successful AI implementations in preoperative assessment (American Society of Anesthesiologists Physical Status (ASA-PS) prediction with accuracy comparable to anesthesiologists), intraoperative monitoring (hypotension prediction 15 minutes in advance), and postoperative risk stratification (MySurgeryRisk system [15] for predicting complications) [12]. However, these applications typically focus on specific, well-defined tasks rather than the comprehensive knowledge assessment required for board-style examinations.

Technical and methodological considerations

The development of our standardized evaluation framework addresses several methodological concerns identified in the broader literature. Lee et al. (2024) noted that most LLM medical evaluation studies used 50 or fewer questions (64.3%) and that only 14.7% employed prompt engineering techniques [11]. Our study utilized 100 questions with standardized prompts, representing a more comprehensive evaluation approach. The framework's modular design allows for adaptation to other specialties and question formats, potentially facilitating broader research in subspecialty AI evaluation.

Our API-based evaluation approach minimizes potential bias while ensuring reproducible results, addressing concerns about methodological consistency in AI medical evaluation. The caching mechanism reduces computational costs and enables detailed analysis of response patterns and consistency, providing insights into model behavior that extend beyond simple accuracy metrics.

Several limitations should be considered when interpreting these results within the broader context of AI medical evaluation. While comprehensive, our 100-question sample may not capture the full breadth of cardiothoracic anesthesia knowledge, and the questions were explicitly developed for this study rather than validated board examination content. Current LLM APIs do not support direct image processing, which limits our evaluation of visual interpretation skills critical to the subspecialty.

AI models undergo continuous updates, and performance may vary with different model versions, though our standardized approach ensures consistency within this evaluation timeframe. Alternative prompt formats might yield different performance levels, but our standardized approach prioritized consistency over optimization, aligning with recommendations for reproducible AI evaluation methodologies. Additionally, the study evaluated isolated knowledge assessment rather than integrated clinical decision-making in real-world scenarios, which may not fully capture the complexity of clinical practice where contextual factors and patient-specific considerations play crucial roles.

Future directions and research priorities

Several critical research priorities emerge from our findings and the broader literature. As LLMs develop image processing capabilities, comprehensive evaluation including visual interpretation will be essential for subspecialties heavily dependent on visual data interpretation. Banerji et al. (2025) emphasized that multimodal clinical AI must be fully attuned to the particular challenges and constraints of the clinic, requiring clinician involvement throughout development rather than just at deployment [13].

Investigating whether subspecialty-specific training data can improve performance in specialized domains represents a critical research priority. The establishment of actively managed multidisciplinary communities, as recommended by Banerji et al., could facilitate collaborative development of AI tools that effectively augment rather than replace subspecialty expertise [13]. Such communities would ensure that AI development remains grounded in clinical reality and addresses the specific needs of subspecialty practice.

Recommendations for clinical practice and policy

Based on these findings and the broader literature, several recommendations emerge for healthcare organizations and practitioners. Healthcare organizations should exercise caution when implementing LLMs for clinical decision support in subspecialty domains, ensuring that subspecialty-specific validation is mandatory before deploying AI tools in specialized clinical areas. This recommendation aligns with emerging best practices that emphasize the need for robust validation frameworks specifically designed for medical subspecialties.

LLMs may be better suited for educational support rather than direct clinical decision-making in their current form, though regular assessment of LLM performance should accompany any clinical implementation. Future LLM development should prioritize multimodal capabilities essential for medical subspecialties, particularly those requiring integration of visual and textual information. Healthcare practitioners should maintain appropriate skepticism regarding AI capabilities in specialized domains while remaining open to future developments, recognizing that the goal should be developing AI tools that effectively augment rather than replace subspecialty expertise when supported by adequate validation and appropriate limitations.

The establishment of multidisciplinary communities involving clinicians throughout the AI development process, as recommended by Banerji et al., represents a critical step toward ensuring that AI tools are appropriately designed for clinical subspecialty applications [13]. Such collaborative approaches could help bridge the gap between technological capability and clinical utility, ultimately leading to more effective and trustworthy AI tools for subspecialty medicine.

## Conclusions

This study demonstrates that current large language models, while impressive in their general medical knowledge capabilities, show significant limitations when applied to subspecialty-level cardiothoracic anesthesia content. Both models performed well below levels considered acceptable for clinical decision support, highlighting the critical distinction between broad medical knowledge and subspecialty expertise. The poor performance on image-based questions further emphasizes the limitations of current text-only LLM interfaces for subspecialties requiring extensive visual data interpretation, such as electrocardiographic analysis and device programming that are fundamental to cardiothoracic anesthesia practice.

These findings highlight the necessity of rigorous subspecialty-specific validation before integrating LLMs into high-stakes clinical environments such as cardiothoracic anesthesia. Our data show that both Claude and ChatGPT-4 fall significantly short of the accuracy required for safe decision support in this domain, reinforcing the importance of involving clinicians with domain expertise throughout the AI development and evaluation process, not just at the point of deployment. While LLMs may have potential in educational settings or broader medical use cases, their application in subspecialty clinical reasoning, particularly for complex, image-based, or nuanced cardiothoracic anesthesia scenarios, remains premature with current capabilities. The reproducible framework developed in this study offers a structured approach to assessing LLM performance within specialized areas of medicine. As models continue to evolve, especially with the anticipated integration of multimodal functionality, periodic reassessment using standardized tools like ours will be essential to determine when, if ever, these technologies meet the threshold for clinical utility. Until then, healthcare systems and clinicians should approach the use of LLMs in cardiothoracic anesthesia with cautious optimism, recognizing that the goal is to augment, not replace, expert judgment, anchored in validated, specialty-specific performance benchmarks.

## Appendices

Appendix A: technical implementation details

A.1 System Requirements

- Python 3.9 or higher

- Required libraries: pandas (1.5.0+), requests (2.28.0+), matplotlib (3.6.0+), seaborn (0.12.0+)

- API access to Claude (Anthropic) and ChatGPT-4 (OpenAI)

- Minimum 4GB RAM for data processing

A.2 Installation Instructions

```bash

# Create virtual environment

python3 -m venv llm_evaluation_env

source llm_evaluation_env/bin/activate

# Install required packages

pip install pandas>=1.5.0 requests>=2.28.0 matplotlib>=3.6.0 seaborn>=0.12.0

# Clone repository (if applicable)

git clone [repository_url]

cd cardiothoracic_llm_comparison_project

A.3 Configuration Setup

```python

# Example configuration in data_processing.py

CONFIG = {

    "data_dir": "./data",

    "results_dir": "./results", 

    "questions_file": "./data/questions.txt",

    "answers_file": "./data/correct_answers.csv",

    "claude_api_key": "YOUR_CLAUDE_API_KEY",

    "gpt4_api_key": "YOUR_GPT4_API_KEY",

    "rate_limit_delay": 1.0  # seconds between API calls }

Appendix B: technical implementation details

**Table 4:** System Architecture and Implementation Specifications

Component	Technology Stack	Function	Performance MEtrics
Data Processing Module			
Language	Python 3.9	Question parsing, API management	100% success rate
Libraries	pandas, json, requests	Data handling and API calls	<1s response time
Caching	Local file system	Response storage	100% reliability
Analysis Module			
Statistical Analysis	scipy.stats	Accuracy calculations, significance testing	All tests completed
Data Visualization	matplotlib, seaborn	Performance charts, comparison plots	All figures generated
Reporting	Custom scripts	Automated report generation	Complete coverage
API Integration			
Claude API	Anthropic Claude-3	Question evaluation	100% uptime
ChatGPT-4 API	OpenAI GPT-4	Question evaluation	100% uptime
Rate Limiting	1 request/second	Compliance with API limits	No violations
Quality Assurance			
Prompt Standardization	Fixed template	Consistent question presentation	100% compliance
Response Validation	Automated checking	Answer format verification	100% valid responses
Reproducibility	Seed setting, caching	Consistent results	100% reproducible

Appendix C: sample questions

**Table 5:** Sample Questions and LLM Responses (Representative Subset) Note: This table represents a subset of the complete 100-question evaluation. Full dataset available upon request.

Question ID	Category	Question text (abbreviated)	Correct answer	Claude response	ChatGPT-4 response	Both correct
Q001	Electrophysiology	70-year-old with palpitation, narrow complex tachycardia 160 bpm, no clear P waves...	B	B ✓	C ✗	No
Q002	Pacemaker Programming	Patient with dual-chamber pacemaker, AV delay optimization for...	A	A ✓	A ✓	Yes
Q003	Perioperative Complications	Post-cardiac surgery patient develops sudden hypotension with...	C	B ✗	C ✓	No
Q004	Antiarrhythmic Pharmacology	Amiodarone loading dose calculation for 80kg patient with...	D	D ✓	B ✗	No
Q005	Mechanical Support	IABP timing optimization in patient with severe LV dysfunction...	B	C ✗	B ✓	No
Q006	Electrophysiology	Wide complex tachycardia differential diagnosis based on...	A	A ✓	D ✗	No
Q007	Pacemaker Programming	Rate-responsive pacing parameters for active 65-year-old...	C	B ✗	C ✓	No
Q008	Perioperative Complications	Management of refractory ventricular fibrillation during...	B	B ✓	B ✓	Yes
Q009	Antiarrhythmic Pharmacology	Drug interaction between warfarin and amiodarone...	D	C ✗	D ✓	No
Q010	Mechanical Support	ECMO weaning criteria for post-cardiotomy patient...	A	B ✗	A ✓	No

Appendix D: statistical analysis code

C.1 Accuracy Calculation

```python

def calculate_accuracy(responses, correct_answers):

    """Calculate accuracy percentage for LLM responses."""

    correct_count = sum(1 for i, response in enumerate(responses) 

                       if response == correct_answers[i])

    accuracy = (correct_count / len(responses)) * 100

    return accuracy, correct_count

def calculate_confidence_interval(correct, total, confidence=0.95):

    """Calculate binomial confidence interval."""

    from scipy import stats

    return stats.binom.interval(confidence, total, correct/total)

```

C.2 Statistical Comparison

```python

def compare_accuracies(claude_correct, claude_total, gpt4_correct, gpt4_total):

    """Compare accuracy between two models using Fisher's exact test."""

    from scipy.stats import fisher_exact

    # Create contingency table

    table = [[claude_correct, claude_total - claude_correct],

             [gpt4_correct, gpt4_total - gpt4_correct]]

        odds_ratio, p_value = fisher_exact(table)

    return odds_ratio, p_value

```

Appendix E: statistical analysis results

**Table 6:** Comprehensive Statistical Analysis *Statistically significant (p < 0.05)

Metric	Value	95% CI	Statistical Test	p value
Overall Performance Comparison				
Claude Accuracy	32.0%	23.1% - 41.8%	Fisher's Exact Test	0.042*
ChatGPT-4 Accuracy	23.0%	15.2% - 32.4%	Fisher's Exact Test	0.042*
Difference	+9.0%	+0.3% - +17.7%	Fisher's Exact Test	0.042*
Inter-Model Agreement				
Cohen's Kappa	0.23	0.08 - 0.38	Kappa Statistic	<0.001*
Agreement Rate	58.0%	47.8% - 67.8%	Proportion Test	-
Category-Specific Analysis				
Electrophysiology Difference	+8.6%	-5.2% - +22.4%	Fisher's Exact Test	0.218
Pacemaker Programming Difference	+12.0%	-7.1% - +31.1%	Fisher's Exact Test	0.214
Complications Difference	+10.0%	-11.9% - +31.9%	Fisher's Exact Test	0.367
Pharmacology Difference	+8.3%	-18.1% - +34.7%	Fisher's Exact Test	0.542
Mechanical Support Difference	0.0%	-35.4% - +35.4%	Fisher's Exact Test	1.000

Appendix F: question categories and distribution 

**Table 7:** Category-Specific Performance Metrics

Category	Total Questions	Claude Correct	Claude %	ChatGPT-4 Correct	ChatGPT-4 %	Difficulty Level *	Common Error Type
Cardiac Electrophysiology	35	12	34.3%	9	25.7%	High	Rhythm interpretation, conduction disorders
Pacemaker Programming	25	9	36.0%	6	24.0%	High	Parameter optimization, troubleshooting
Perioperative Complications	20	6	30.0%	4	20.0%	Moderate	Emergency management, drug dosing
Antiarrhythmic Pharmacology	12	3	25.0%	2	16.7%	High	Drug interactions, dosing calculations
Mechanical Support Devices	8	2	25.0%	2	25.0%	Very High	Device programming, troubleshooting
Overall	100	32	32.0%	23	23.0%	High	Concept integration, visual interpretation
